# Global Results of Implantable Loop Recorder for Detection of Atrial Fibrillation After Stroke: Reveal LINQ Registry

**DOI:** 10.1161/JAHA.124.035956

**Published:** 2024-10-25

**Authors:** Kazunori Toyoda, Kengo Kusano, Yasuyuki Iguchi, Takanori Ikeda, Itsuro Morishima, Hirofumi Tomita, Taku Asano, Teiichi Yamane, Ichiro Nakahara, Eiichi Watanabe, Junjiroh Koyama, Ritsushi Kato, Hiroshi Morita, Teruyuki Hirano, Kyoko Soejima, Shingen Owada, Haruhiko Abe, Masahiro Yasaka, Toshihiro Nakamura, Scott Kasner, Andrea Natale, Sean Beinart, Alpesh N. Amin, Erika Pouliot, Noreli Franco, Kazuhiro Hidaka, Ken Okumura

**Affiliations:** ^1^ Department of Cerebrovascular Medicine National Cerebral and Cardiovascular Center Suita Japan; ^2^ Department of Cardiovascular Medicine National Cerebral and Cardiovascular Center Suita Japan; ^3^ Department of Neurology The Jikei University School of Medicine Tokyo Japan; ^4^ Department of Cardiovascular Medicine Toho University Faculty of Medicine Tokyo Japan; ^5^ Department of Cardiology Ogaki Municipal Hospital Ogaki Japan; ^6^ Department of Cardiology and Nephrology Hirosaki University Graduate School of Medicine Hirosaki Japan; ^7^ Division of Cardiology Showa University School of Medicine Tokyo Japan; ^8^ Division of Cardiology The Jikei University School of Medicine Tokyo Japan; ^9^ Department of Neurosurgery Fujita Health University Bantane Hospital Nagoya Japan; ^10^ Department of Internal Medicine Fujita Health University Bantane Hospital Nagoya Japan; ^11^ Division of Cardiology Saiseikai Kumamoto Hospital Kumamoto Japan; ^12^ Department of Cardiology Saitama Medical University, International Medical Center Hidaka Japan; ^13^ Department of Cardiovascular Therapeutics Okayama University Graduate School of Medicine Okayama Japan; ^14^ Department of Stroke and Cerebrovascular Medicine Kyorin University School of Medicine Tokyo Japan; ^15^ Department of Cardiology Kyorin University School of Medicine Tokyo Japan; ^16^ Division of Cardiology, Department of Internal Medicine Iwate Medical University School of Medicine Shiwa Japan; ^17^ Department of Heart Rhythm Management University of Occupational and Environmental Health Kitakyushu Japan; ^18^ Department of Cerebrovascular Medicine Fukuoka Neurosurgical Hospital Fukuoka Japan; ^19^ Department of Cardiology National Hospital Organization Kyushu Medical Center Fukuoka Japan; ^20^ Comprehensive Stroke Center, Department of Neurology University of Pennsylvania Philadelphia PA USA; ^21^ Department of Biomedicine and Prevention, Division of Cardiology University of Tor Vergata Rome Italy; ^22^ Center for Cardiac and Vascular Research Washington Adventist Hospital Takoma Park MD USA; ^23^ Department of Medicine University of California Irvine CA USA; ^24^ Cardiac Rhythm Management, Clinical Department Medtronic Inc. Minneapolis MN USA; ^25^ Japan Clinical and Medical Affairs Medtronic Japan Co., Ltd. Tokyo Japan

**Keywords:** anticoagulation, atrial fibrillation, cardioembolism, cerebral infarction, cryptogenic ischemic stroke, electrocardiographic monitoring, Ischemic Stroke, Atrial Fibrillation

## Abstract

**Background:**

We aimed to quantify the incidence of atrial fibrillation (AF) in patients with cryptogenic stroke globally, as well as separately in patients in and outside of Japan, using an implantable loop recorder from a prospective, observational, Reveal LINQ Registry.

**Methods and Results:**

Patients developing cryptogenic stroke and monitored by implantable loop recorder for searching AF were studied. The primary end point was incidence of AF within 36 months after insertion. Secondary end points were recurrent ischemic stroke/transient ischemic attack and AF‐related treatment strategies. A total of 271 patients (61.6±14.3 years, 170 men, 60 from Japan) were enrolled from 12 countries. AF was detected in 28.2% at 36 months. The median time from enrollment to AF detection was 7.9 months. During the first 12 months, the AF detection rate slope was relatively steeper in the Japanese subgroup versus non‐Japanese patients. However, by 3 years, the cumulative incidence of AF detection did not differ between groups. Age was the only variable associated with AF detection (hazard ratio, 1.05 [95% CI, 1.02–1.07] per year), trending higher in older age groups. Of the 271 patients, 11 (4.1%) developed recurrent ischemic stroke/transient ischemic attack; AF was detected by implantable loop recorder in only 1 of these patients. Patients with detected AF were more commonly taking oral anticoagulation than those without AF at the last follow‐up (64.7% versus 25.3%, *P*<0.001).

**Conclusions:**

The rate of AF detection was similar to other studies in stroke populations monitored by implantable loop recorders, including CRYSTAL‐AF (Cryptogenic Stroke and Underlying Atrial Fibrillation), STROKE‐AF (Stroke of Unknown Cause and Underlying Atrial Fibrillation) and PER‐DIEM (Post‐Embolic Rhythm Detection With Implantable Versus External Monitoring). Patients with detected AF more commonly initiated anticoagulation than those without AF.

**Nonstandard Abbreviations and Acronyms**
ARTESIA: Apixaban for the Reduction of Thrombo‐Embolism in Patients with Device‐Detected Subclinical Atrial Fibrillation
ASSERT: Asymptomatic Atrial Fibrillation and Stroke Evaluation in Pacemaker Patients and the Atrial Fibrillation Reduction Atrial Pacing Trial
ASSERT‐II: Prevalence of Subclinical Atrial Fibrillation Using an Implantable Cardiac Monitor
CRYSTAL‐AF: Cryptogenic Stroke and Underlying Atrial Fibrillation
ESUS: embolic stroke of undetermined source
ILR: implantable loop recorder
NAVIGATE ESUS: New Approach Rivaroxaban Inhibition of Factor Xa in a Global Trial Versus ASA to Prevent Embolism in Embolic Stroke of Undetermined Source
OAC: oral anticoagulation
PER‐DIEM: Post‐Embolic Rhythm Detection With Implantable Versus External Monitoring
RE‐SPECT ESUS: Randomized, Double‐Blind Evaluation in Secondary Stroke Prevention Comparing the Efficacy and Safety of the Oral Thrombin Inhibitor Dabigatran Etexilate Versus Acetylsalicylic Acid in Patients With Embolic Stroke of Undetermined Source
REVEAL‐AF: Incidence of AF in High‐Risk Patients
STROKE‐AF: Stroke of Unknown Cause and Underlying Atrial Fibrillation

**Clinical Perspective**

**What Is New?**

- The 3‐year cumulative incidence of atrial fibrillation detection in patients with cryptogenic stroke using implantable loop recorders did not differ between patients in Japan and those in other countries, although the slope of detection rate was relatively steeper in the Japanese subgroup for the first 12 months after implant.
- Age was the only variable associated with detection of atrial fibrillation, and the rate differed among different age groups.

**What Are the Clinical Implications?**

- Detection of atrial fibrillation could change treatment strategies for patients with cryptogenic stroke, such as oral anticoagulation, and thereby have an impact on preventing stroke recurrence.

Atrial fibrillation (AF) is often covert in patients with stroke.^1^ The development of long‐term electrocardiographic monitoring, including implantable loop recorder (ILR), has made stroke neurologists more interested in searching for covert AF in patients with cryptogenic stroke, which lacks a clearly definable pathogenetic mechanism and is often embolic.^2^, ^3^ In the 3 major randomized controlled trials using ILR monitoring in patients with ischemic stroke, AF was detected in ≈12% to 15% of patients by 12 months after ILR implant.^4^, ^5^, ^6^ Meta‐analyses on poststroke cardiac rhythm monitoring have shown higher rates of AF detection and oral anticoagulation (OAC) initiation in patients with ischemic stroke monitored with ILR compared with those receiving Holter monitors or external loop recorders.^7^, ^8^ In recent European guidelines, prolonged cardiac monitoring instead of standard 24‐hour monitoring was recommended and the use of ILR instead of nonimplantable devices was suggested to increase AF detection for adult patients with ischemic stroke or transient ischemic attack (TIA) of undetermined origin.^9^ Long‐term rhythm monitoring using ILR was described as reasonable to improve AF detection for patients with stroke or TIA in recent US guidelines.^10^

The prevalence of AF in Asia was reportedly lower than in Western countries, although it has been increasing due to aging populations.^11^ In ASSERT (Asymptomatic Atrial Fibrillation and Stroke Evaluation in Pacemaker Patients and the Atrial Fibrillation Reduction Atrial Pacing Trial), Black Africans, Chinese, and Japanese older patients with hypertension using implanted devices had a lower incidence of AF compared with Europeans during a 2.5‐year observation period.^12^ It remains unknown whether differences in the AF detection rate exist among patients with cryptogenic stroke from different countries or races. The AF detection rate using ILRs in Japan was reportedly relatively high (21.4% at 3 months) in a multicenter registry of patients with cryptogenic stroke.^13^ Here, we aimed to quantify the incidence of AF in patients with cryptogenic stroke globally, as well as separately in patients in and outside of Japan, using an ILR from a prospective, observational, Reveal LINQ Registry.^14^ We also assessed recurrence of ischemic stroke/TIA, as well as AF‐related treatment strategies.

## Methods

### Study Design and Setting

The Reveal LINQ Registry was a prospective, nonrandomized, single‐arm, open label, observational registry (ClinicalTrials.gov; NCT02746471), involving 12 countries, aiming to characterize the role of Reveal LINQ ILR (Medtronic Inc., Minneapolis, MN) in the patient care pathway.^14^ The study was designed and conducted in accordance with the Declaration of Helsinki and followed the Strengthening the Reporting of Observational Studies in Epidemiology reporting guideline. The local institutional review boards or ethics committees approved the study protocol at each participating center, and all patients provided written informed consent. The data that support the findings of this study are available from the corresponding author upon reasonable request.

### Participants

Patients were included in the present analysis if (1) they had a history of cryptogenic stroke or TIA on the basis of the criteria by the Cryptogenic Stroke/Embolic Stroke of Undetermined Source (ESUS) International Working Group,^3^ (2) the reason for monitoring with an ILR was cryptogenic stroke, (3) they had available device data, and (4) they had a minimum of 1 contact with their care provider after ILR insertion (to allow for collection of any treatment strategies after implant). Patients with a history of AF or atrial ablation were excluded.

### Study Procedures

The ILR insertion procedure for this registry has been previously described.^14^, ^15^ AF was defined as an episode ≥2 minutes retrospectively adjudicated by 1 or 2 independent reviewers unrelated to the study (Medtronic monitoring center). AF status was also based on diagnoses made by treating physicians during follow‐up and recorded in case report forms. The AF detection algorithm operates through continuous assessment of the regularity of RR intervals within a 2‐minute time window to recognize AF. Data were collected at in‐office clinic visits, review of remote monitor transmissions, or chart review for reportable events. Follow‐up was required at a minimum every 6 months ±3 months after insertion, and real‐time reporting was performed for treatment strategies and adverse events, including stroke, TIA, OAC, and cardiac device therapies. Patients were monitored with the ILR for up to 36 months from time of insertion. When the registry started data collection in March 2014 and through December 2017, sites were required to follow patients for a minimum of 18 months. In September 2016, the required follow‐up was extended to 36 months. All patients from Portugal and Japan were enrolled after that date and were thus followed for the extended follow‐up period.

### End Points

The primary end point of this analysis was the incidence of adjudicated AF lasting ≥2 minutes within 36 months after ILR insertion. Secondary end points were recurrent ischemic stroke/TIA, recurrent ischemic stroke, and AF‐related treatment strategies, including OAC therapy and cardiac device therapies. For the treatment strategies analysis, if patients had no AF, they were required to have a minimum of 1 year of follow‐up data to allow for enough time to detect AF and record any treatment strategies. End points were assessed both overall and separately in patients from Japan and those outside Japan.

### Statistical Analysis

**Figure 1:**
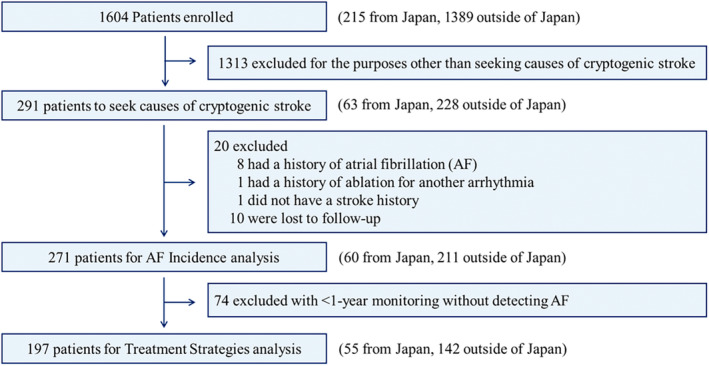
Study flowchart.

Statistical analyses were performed using SAS version 9.4 (SAS Institute, Cary, NC). All analyses were focused on comparing patients from Japan versus patients not from Japan. The analysis data sets consisted of 2 different cohorts (AF Incidence analysis and Treatment Strategies analysis), the second being a subset of the first (Figure 1). The first cohort was used for baseline, time to recurrent stroke analyses, time to first AF detection, and predictors of AF analysis. The second cohort was used for treatment strategy reporting.

Patients were analyzed post hoc according to their country of implant (Japan versus non‐Japan). χ^2^ tests were used when testing categorical variables for independence, provided sample sizes were sufficient. When sample sizes were too small, Fisher's exact test was used instead. *T* tests were used when comparing normally distributed continuous variables. The Wilcoxon rank‐sum exact test was used when comparing nonparametrically distributed continuous variables.

Survival estimates are reported as well as a hazard ratio estimate for the effect with corresponding 2‐sided 95% CIs. A limit of 20 patients remaining at risk, per cohort, was used to censor Kaplan–Meier plots for both time to the first detection of AF and cumulative incident rate of ischemic stroke/TIA events. For the plot showing time to the first detection of AF according to age grouping, the plot was not censored on the basis of the number of patients remaining at risk. A multivariable Cox proportional hazard model analyzed time to first AF episode. When time‐to‐event methods were used, only participants with no follow‐up time from randomization had missing data. There is potential for type I error due to multiple comparisons, thus findings should be interpreted as exploratory. Statistical significance was set at a 2‐sided *P* value of 0.05 for all analyses.

## Results

Participants were enrolled from March 2014 to January 2018 at 51 study sites located in 12 countries (United States, Japan, Italy, Portugal, Saudi Arabia, Netherlands, Belgium, Greece, Germany, Spain, Israel, and United Kingdom [in descending order of registration number]). The last patient completed follow‐up in May 2021. Of the 1604 patients who participated in the registry, 291 were enrolled for the purpose of detecting covert AF as a pathogenetic cause of cryptogenic stroke from 37 sites in 12 countries (Figure 1). Twenty of these 291 patients were excluded from the present analyses due to a history of AF, a history of ablation for another arrhythmia, the lack of a stroke history, and no follow‐up data after ILR insertion. Thus, 271 patients (61.6±14.3 years, 170 men) were studied. There were no unexpected battery depletions reported during the study period. Of the 72 patients who remained in the study for ≥36 months, 1 had a battery depletion. Median time of study follow‐up was 20.0 months (interquartile range [IQR], 8.0–37.3).

### AF Incidence Analysis

Table 1 shows patient baseline characteristics. The median CHA_2_DS_2_‐VASc score was 4 (IQR, 3–5), and the median CHADS_2_ score was 3 (IQR, 4–6). The median time from the most recent stroke event to ILR insertion was 1.8 months (IQR, 0.6–4.3).

**Table 1:** Baseline Characteristics of Patients

Characteristic	All (n=271)	Japanese (n=60)	Non‐Japanese§ (n=211)
Age, y	61.6±14.3	59.1±15.7	62.3±13.9
Male sex	170 (62.7)	46 (76.7)	124 (58.8)*
Race			‡
White	129 (47.6)	0 (0.0)	129 (61.1)
Asian	64 (23.6)	60 (100.0)	4 (1.9)
Black	13 (4.8)	0 (0.0)	13 (6.2)
Other	2 (0.7)	0 (0.0)	2 (0.9)
Not reportable per local law or regulations	63 (23.2)	0 (0.0)	63 (29.9)
Body mass index	27.4±6.0	22.9±2.9	28.7±6.0^‡^
Medical history			
Diabetes	54 (19.9)	8 (13.3)	46 (21.8)
Myocardial infarction	9 (3.3)	0 (0.0)	9 (4.3)
Hypertension	128 (47.2)	21 (35.0)	107 (50.7)*
Congestive heart failure	10 (3.7)	3 (5.0)	7 (3.3)
Structural heart disease	5 (1.8)	1 (1.7)	4 (1.9)
Peripheral vascular disease	5 (1.8)	0 (0.0)	5 (2.4)
Aortic plaque	5 (1.8)	0 (0.0)	5 (2.4)
CHA_2_DS_2_‐VASc score	4 (3–5)	3 (2–4)	4 (3–5)^†^
CHADS_2_ score	3 (4–6)	3 (2–3)	3 (2–4)*
Intrinsic QRS duration, ms	95.3±22.5	97.9±21.3	94.4±22.9
PR interval, ms	177±78	198±142	169±28
Medications			
Any anticoagulants	57 (21.1)	26 (43.3)	31 (14.8)^‡^
Warfarin	26 (9.6)	14 (23.3)	12 (5.7)
Direct oral anticoagulants	23 (8.5)	11 (18.3)	12 (5.7)
Unknown	8 (3.0)	1 (1.7)	7 (3.3)
Antiplatelets	181 (67.3)	27 (45.0)	154 (73.7)^‡^
Antiarrhythmic agents	5 (1.9)	1 (1.7)	4 (1.9)
β blockers	76 (28.3)	3 (5.0)	73 (34.9)^‡^
ACE inhibitors	74 (27.5)	6 (10.0)	68 (32.5)^‡^
Time from most recent cryptogenic stroke/TIA to ILR implant, mo	1.8 (0.6–4.3)	1.0 (0.6–2.8)	2.3 (0.7–4.7)

Data are n (%), mean±SD, or median (interquartile range). Twenty‐three patients (4 from Japan) are missed for “body mass index.” Seventy patients (8 from Japan) are missed for intrinsic QRS duration. Seventy‐six patients (8 from Japan) are missed for PR interval. One patient (non‐Japanese) is missed for anticoagulant medication. Two patients (both non‐Japanese) are missed for other medications. A total of 219 patients (48 from Japan) are missed for time from the stroke event to ILR implant. ACE indicates angiotensin‐converting enzyme; ILR, implantable loop recorder; and TIA, transient ischemic attack. One is Native Hawaiian or other Pacific Islander. The detailed information is not entered in the other.

*P<0.05, ^†^ *P*<0.01, ^‡^ *P*<0.001 vs “Japan.”

^§^ 132 from United States, 24 from Italy, 16 from Greece, 10 from Saudi Arabia, 8 each from Germany and Spain, 7 from Israel, 3 from Belgium, 2 from Netherlands, and 1 from Portugal.

**Figure 2:**
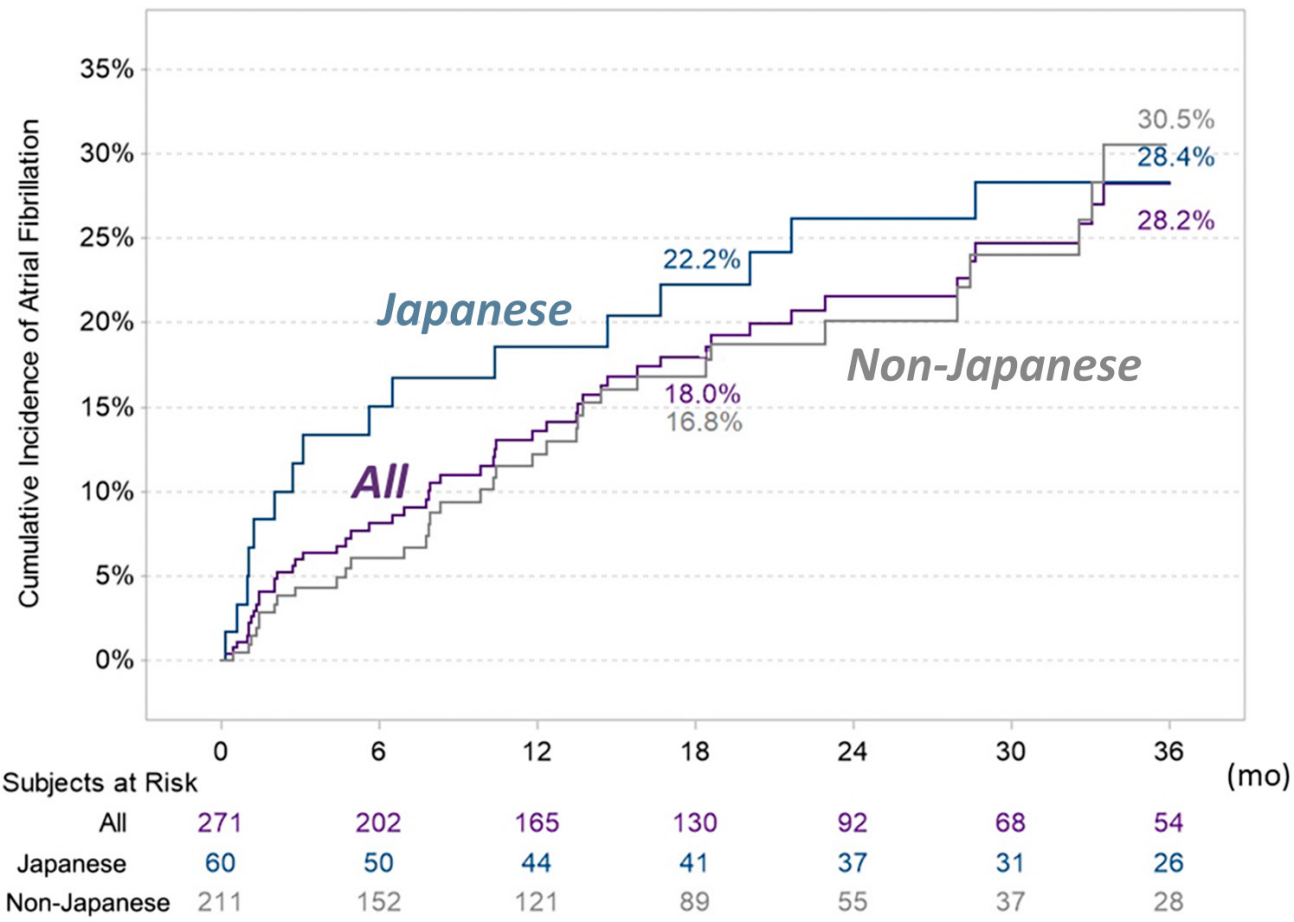
Time to the first detection of atrial fibrillation.

Figure 2 shows the time to the first AF detection after ILR insertion. AF was detected in 1.5% (95% CI, 0.6%–3.9%) of patients at 1 month, 6.0% (95% CI, 3.7%–9.6%) at 3 months, 13.6% (95% CI, 9.7%–18.7%) at 12 months, 18.0% (95% CI, 13.4%–23.8%) at 18 months, and 28.2% (95% CI, 21.6%–36.4%) at 36 months. The median time from enrollment to AF detection was 7.9 months (IQR, 2.0–15.8).

### Comparison Between Japanese and Non‐Japanese Subgroups

The Japanese subgroup included 60 patients monitored for a median duration of 37.9 (IQR, 33.1–39.0) months. The non‐Japanese subgroup included 211 patients monitored for a shorter period for reasons described above, a median of 17.5 (IQR, 6.0–29.5) months. Several differences were observed when comparing these subgroups, including a higher proportion of Asians, and men; lower BMI; lower proportion of patients with a history of hypertension; lower CHADS_2_ and CHA_2_DS_2_‐VASc scores; higher proportion of patients on baseline OAC; and lower proportion of patients on baseline antiplatelets, β blockers, and angiotensin‐converting enzyme inhibitors in the Japanese versus non‐Japanese patients (Table 1). Before ILR insertion, the Japanese subgroup had more imaging tests of head/neck performed, including magnetic resonance imaging scans, magnetic resonance angiography, computed tomography scan, and Doppler of carotids/intracerebral arteries than the non‐Japanese subgroup (Table 2). As for cardiac monitoring, ECG Holter, transthoracic echocardiography, and transesophageal echocardiography were more often performed in the Japanese subgroup.

**Table 2:** Diagnostics Tests Before Insertion of Implantable Loop Recorders

Characteristic	All (n=271)	Japanese (n=60)	Non‐Japanese (n=211)
Head and neck			
CT	143 (52.8)	47 (78.3)	96 (45.5)^‡^
MR imaging	168 (62.0)	57 (95.0)	111 (52.6)^‡^
CT angiography	63 (23.2)	19 (31.7)	44 (20.9)
MR angiography	94 (34.7)	55 (91.7)	39 (18.5)^‡^
Carotid/transcranial Doppler	90 (33.2)	32 (53.3)	58 (27.5)^‡^
Cardiac monitoring			
Holter ECG	106 (39.1)	51 (85.0)	55 (26.1)^‡^
External loop/event recorder	18 (6.6)	2 (3.3)	16 (7.6)
ECG basic/telemetric	41 (15.1)	9 (15.0)	32 (15.2)
Transthoracic echocardiography	163 (60.1)	58 (96.7)	105 (49.8)^‡^
Transesophageal echocardiography	101 (37.3)	42 (70.0)	59 (28.0)^‡^

Data are n (%). CT indicates computed tomography; and MR, magnetic resonance.

^‡^ *P*<0.001 vs “Japan.”

No statistically significant difference in the incidence of AF detection was observed between subgroups (*P*=0.58; Figure 2). The slope of AF detection rate was relatively steeper in the Japanese subgroup for the first 12 months; the rate of AF detection at 4 months in the Japanese subgroup was >3‐fold higher than the non‐Japanese subgroup. However, cumulative incidence rates leveled off by 36 months. As a straight percentage (ignoring differences in follow‐up times), AF was detected in 16 patients (27%) in the Japanese subgroup and 35 (17%) in the non‐Japanese subgroup. The median time from insertion to AF detection was 4.4 (IQR, 1.1–15.7) months for the Japanese and 9.8 (IQR, 2.8–15.8) months for the non‐Japanese subgroups (*P*=0.20).

The 51 patients with AF detection had a total of 513 AF episodes with a median of 3 episodes per patient (Table 3). The median duration of the longest single AF episode per patient was 34 (IQR, 6–514) minutes.

**Table 3:** Duration and Number of Adjudicated AF

Parameter	All patients with AF (n=51)	Japanese patients with AF (n=16)	Non‐Japanese patients with AF (n=35)
Median duration of longest AF episode, min (IQR)	34 (6–514)	550 (16–1106)	20 (6–354)
Median number of AF episodes (IQR)	3 (1–13)	5.5 (1–15)	3 (1–12)
Number of patients with AF >1 h (%)	23 (45)	10 (63)	13 (37)
Number of patients with AF >6 h (%)	16 (31)	9 (56)	7 (14)
Number of patients with AF >24 h (%)	7 (14)	3 (19)	4 (11)

AF indicates atrial fibrillation; and IQR, interquartile range.

### Predictors of AF

**Figure 3:**
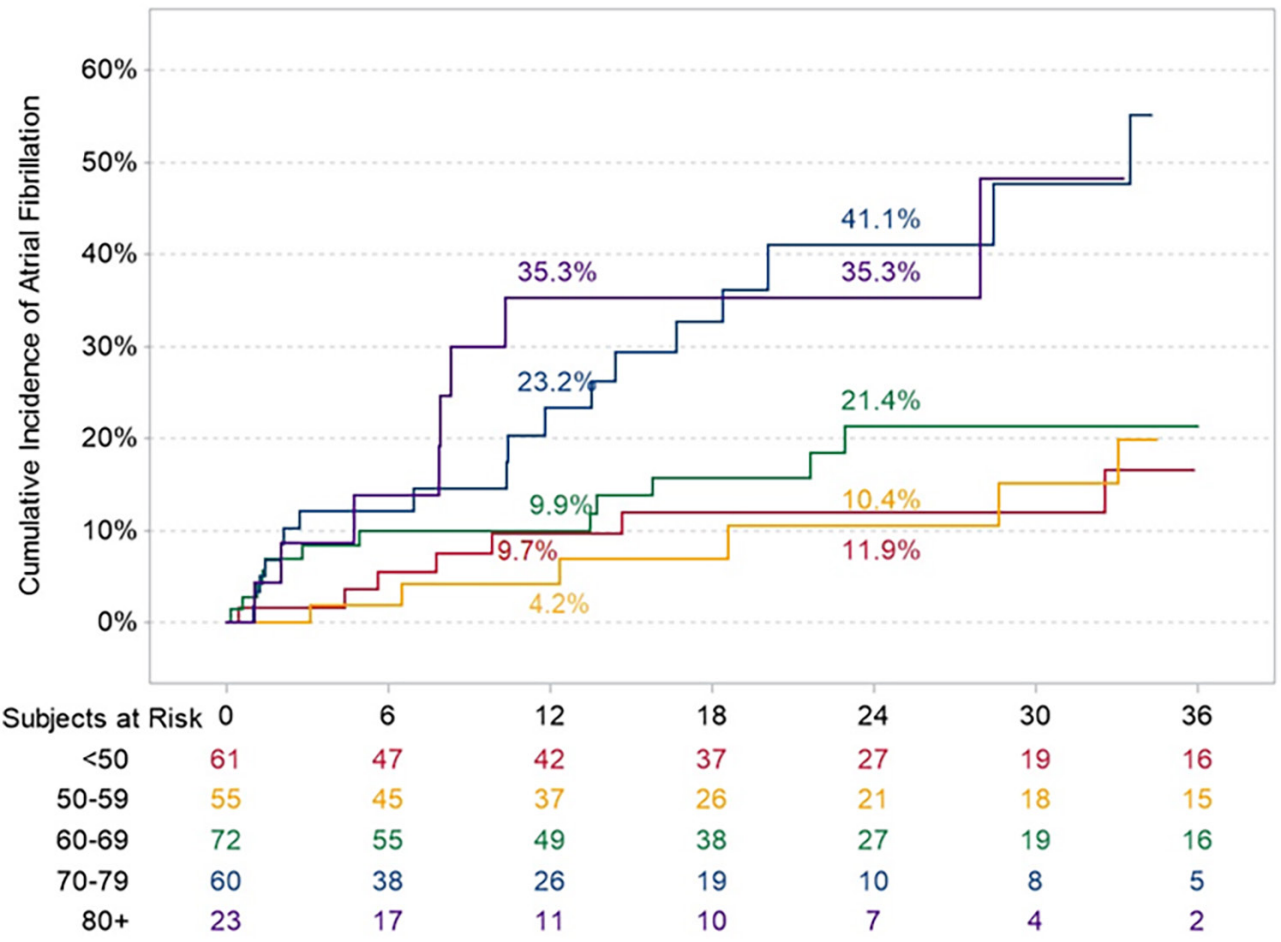
Time to the first detection of atrial fibrillation according to age.

In a multivariable analysis for predictors of AF detection comparing Japanese versus non‐Japanese patients and the components of the CHA_2_DS_2_‐VASc score, only age was associated with an increased likelihood of detecting AF (hazard ratio, 1.05 [95% CI, 1.02–1.07] per year; Table 4). Upon further exploring AF detection according to age group (aged <50 years, 50–59 years, 60–69 years, 70–79 years, and ≥80 yearsr) AF incidence trended higher in older age groups (Figure 3).

**Table 4:** Multivariable Predictors of AF Considering Country and the Components of CHA_2_DS_2_‐VASc

Parameter	Pr>χ^2^	Hazard ratio	Wald 95% CI
Age at implant (per year)	<0.0001	1.05	1.03–1.07
Sex	0.92	0.97	0.54–1.74
Congestive heart failure	0.50	2.00	0.27–14.9
Hypertension	0.09	1.69	0.93–3.07
Diabetes	0.40	1.41	0.64–3.12
Vascular disease*	0.75	0.82	0.24–2.74
Japanese (or not)	0.46	1.27	0.68–2.37

Of the components of CHA_2_DS_2_‐VASc, history of stroke was required for all the patients and was not included in the models. AF indicates atrial fibrillation.

* Vascular disease includes peripheral vascular disease, plaque, and myocardial infarction.

### Recurrent Stroke

**Figure 4:**
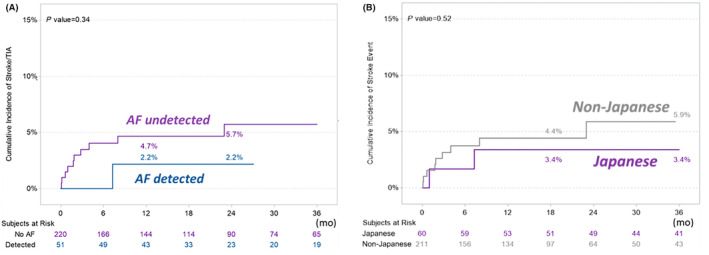
Cumulative incident rate of ischemic stroke/TIA events. (**A**) By presence/absence of subclinical atrial fibrillation; (**B**) by Japanese/non‐Japanese groups. AF indicates atrial fibrillation; and TIA, transient ischemic attack.

Of 271 patients, 11 (4.1%) experienced 15 ischemic stroke/TIA events during follow‐up; 10 of the 11 (3.7%) developed events within the initial 12 months of monitoring. TIA occurred in 5 patients (5 events), cardioembolic stroke in 4 (5 events), atherothrombotic stroke in 2 (3 events), and cryptogenic stroke and lacunar stroke in 1 each (each 1 event). AF was detected in only 1 of the 11 patients who developed recurrent stroke. Figure 4A shows the cumulative incident rate of ischemic stroke/TIA events by presence/absence of subclinical AF (*P*=0.34). Of the 11 patients, 2 (3.4%) were registered from Japan and 9 (4.3%) outside of Japan (*P*=0.52, Figure 4B).

### Treatment Strategies Analysis

To allow for enough time to detect AF and record any treatment strategies related to ILR findings, 74 patients who had no AF, and <1 year of follow‐up data were excluded from the treatment strategies analysis. In total, 197 patients were included (Figure 1). Table 5 shows AF‐related treatments according to subgroup and AF status during follow‐up. Twenty‐three of 38 patients (60.5%) with identified AF initiated OAC treatment during follow‐up, while 9 of 27 (33.3%) without AF discontinued OAC. Finally, 33 (64.7%) patients with AF versus 37 (25.3%) patients without AF were on OAC at their last follow‐up (*P* < 0.001). Catheter ablation was performed in 6 (11.8%) and therapeutic device implant in 6 patients with AF (11.8%). No statistically significant differences were observed between the Japanese and non‐Japanese subgroups.

**Table 5:** Treatment Strategies

Parameter	All (n=197)		Japanese (n=55)		Non‐Japanese (n=142)	
Treatment	No AF (n=146)	AF detected (n=51)	No AF (n=39)	AF detected (n=16)	No AF (n=107)	AF detected (n=35)
ILR monitoring time (mo)	28.1±9.4	25.3±11.4	34.8±6.2	31.4±10.2	25.7±9.2	22.6±10.9
OAC						
Medication at baseline	34/145 (23.4)	12/51 (23.5)	18/39 (46.2)	5/16 (31.3)	16/106 (15.1)	7/35 (20.0)
Initiation during the study*	12/100 (12.0)	23/38 (60.5)	2/22 (9.1)	10/11 (90.9)	10/78 (12.8)	13/27 (48.1)
Discontinuation during the study*	9/27 (33.3)	2/9 (22.2)	5/17 (29.4)	1/5 (20.0)	4/10 (40.0)	1/4 (25.0)
On OAC at the last follow‐up	37 (25.3)	33 (64.7)	15 (38.5)	14 (87.5)	22 (20.6)	19 (54.3)
Catheter ablation	2 (1.4)	6 (11.8)	2 (5.1)†	6 (37.5)	0	0
Electrical cardioversion	0	2 (3.9)	0	1 (6.3)	0	1 (2.9)
Therapeutic device (Pacemaker, ICD, CRT)	4 (2.7)	6 (11.8)	2 (5.1)	1 (6.3)	2 (1.9)	5 (14.3)
ILR explanted during the study	10 (6.8)	8 (15.7)	6 (15.4)	5 (31.3)	4 (2.8)	3 (8.6)

AF indicates atrial fibrillation; CRT, cardiac resynchronization therapy; ICD, implantable cardioverter‐defibrillator; ILR, implantable loop recorder; and OAC, oral anticoagulation.

* Change from initial to last follow‐up with medication noted.

^†^ Two patients with ablations did not have adjudicated AF by the monitoring center but were diagnosed with AF on the study clinical report form.

## Discussion

This study showed global results of the incidence of AF in patients with cryptogenic stroke using ILR from a large international registry. The major finding was that AF was detected in 18.0% at 18 months and 28.2% at 36 months after ILR insertion. In the Japanese subgroup, the rate trended slightly higher compared with the non‐Japanese subgroup but not significantly. Only age was statistically associated with AF detection at a significant level. In addition, patients with detected AF during the observation period tended to develop ischemic stroke or TIA less frequently and to initiate OAC more frequently than those without AF. Detection of AF could change treatment strategies, such as OAC use, and thereby have an impact on stroke recurrence.

ILRs are established devices recording higher detection rates of AF in patients with cryptogenic stroke. Meta‐analyses have demonstrated that the probability of AF detection was 2.78‐ to 5.31‐fold higher using ILRs versus conventional cardiac monitoring.^7^, ^8^ The 12‐month detection rate found in our cohort of patients with cryptogenic stroke (13.6%) was similar to those reported in randomized controlled trials (≈12%–15%)^4^, ^5^, ^6^ and most observational studies (≈12%–16%).^16^, ^17^, ^18^, ^19^, ^20^ At 36 months, the rate of AF detection (28.2%) was also similar to those in the CRYSTAL‐AF (Cryptogenic Stroke and Underlying Atrial Fibrillation; 30.0%) and the STROKE‐AF (Stroke of Unknown Cause and Underlying Atrial Fibrillation; 21.7%) trials.^6^, ^21^ We should be careful of the interpretation of these findings because the detection rate of AF in the nonstroke population was rather high (31.0% and 27.1% at 12 months, respectively) and prior stroke (not always cryptogenic) was not predictive of AF detected by ILR in ASSERT‐II (Prevalence of Subclinical Atrial Fibrillation Using an Implantable Cardiac Monitor) or REVEAL‐AF (Incidence of AF in High‐Risk Patients) studies.^22^, ^23^

Some observational studies in patients with cryptogenic stroke have shown 12‐month AF detection rates as high as 24.1% to 33.3%.^13^, ^24^, ^25^, ^26^ A possible explanation for these higher rates could be the age of patients enrolled, who were 5 to 10 years older than our cohort (mean age, 66.4–71.6 versus 61.6 years). Older age has been associated with increased AF detection^26^, ^27^, ^28^, ^29^, ^30^ and was the only statistically significant predictor of AF in the present study. The rate increased by 5% per 1‐year increase in our analysis and by 91% per 10‐year increase in CRYSTAL‐AF.^29^ In contrast, ILR detected AF in ≈10% of patients aged <60 years in our cohort, similar to the 6% reported for that age group in a recent study. Since AF is strongly age dependent, pathogeneses of stroke should be thoroughly examined for younger patients with cryptogenic stroke before implant of ILR.

Another possible reason for high AF detection rates in some studies may be early ILR insertion (within 1 month) after stroke onset. AF seems to be detected relatively often early after index embolic events.^31^ However, it should be noted that AF detected early after stroke is often neurogenic; the mechanisms include transient loss of autonomic control and short‐lasting inflammatory responses by stroke damage.^32^ Neurogenic AF was associated with insular stroke.^33^ In a meta‐analysis involving 22 566 patients with ischemic stroke or TIA, the risk of recurrent stroke was 26% lower in patients with AF detected early after stroke than in those with AF known before stroke, suggesting that poststroke AF arising from neurogenic factors has a low burden and is associated with a low embolic risk.^34^

Of previous reports with higher AF detection rate, a multicenter observational study from Japan showed a high 3‐month rate of 21.4%; the median time from insertion to AF detection was 31.5 days.^13^ A possible reason for this high rate was the selection of study patients on the basis of the Clinical Guide for the Diagnosis of Cryptogenic Stroke Eligible for ILR in Japan.^35^ The guide recommended stricter diagnostic criteria for eligible patients than those by the cryptogenic stroke/ESUS International Working Group.^3^ These criteria included (1) stroke detected by magnetic resonance imaging that is not a single small‐artery lesion; (2) absence of extracranial/intracranial atherosclerosis causing ≥50% luminal stenosis in arteries supplying the ischemic area; (3) no major‐risk cardioembolic source of embolism; (4) no definite diagnosis of paradoxical embolic stroke; (5) no definite diagnosis of aortogenic embolic stroke; and (6) no other specific cause of stroke identified. To check the criteria, magnetic resonance imaging and magnetic resonance angiography, not computed tomography, should be principally performed for brain imaging to delineate embolic infarct patterns and to exclude intracranial artery stenosis that is popular in Asian population.^36^, ^37^ In the present study, Japan was the only participating country from East Asia. The rate of AF detection at 4 months after ILR insertion in patients from Japan was >3‐fold higher than those from other countries. Time from stroke onset to ILR insertion was only a median of 1.0 month in the Japanese cohort, suggesting high AF detection early after stroke.^31^ In addition, the Japanese cohort very often underwent head magnetic resonance imaging, magnetic resonance angiography, and carotid/transcranial Doppler, probably according to the above clinical guide. Thus, the study participants from Japan may have more purely embolic stroke with covert AF. This hypothesis was also discussed in the Japanese cohort subanalysis of the RE‐SPECT ESUS (Randomized, Double‐Blind Evaluation in Secondary Stroke Prevention Comparing the Efficacy and Safety of the Oral Thrombin Inhibitor Dabigatran Etexilate Versus Acetylsalicylic Acid in Patients With Embolic Stroke of Undetermined Source) trial comparing the efficacy and safety of rivaroxaban with aspirin for the prevention of recurrent stroke in patients with ESUS.^38^, ^39^ Dabigatran was potentially associated with a lower risk of recurrent stroke relative to aspirin in Japanese patients, while no statistically significant difference was shown in non‐Japanese patients. In contrast, stroke of an unknown cause due to incomplete workup seemed to be often included in patients outside of Japan, since they often missed essential diagnostic tests for differentiation of cryptogenic stroke.

Incident ischemic stroke/TIA was infrequent in patients with detected AF in the present study and was numerically less common than in those without detection, although one should note that few events could cause statistical bias. One interpretation could be that, once AF was detected, appropriate AF‐associated stroke prevention, including OAC, catheter ablation, and device therapies, were commonly performed and might have decreased the risk of stroke recurrence. Another possible cause of the low event rate was the short observation period. A different study with similar design but completing 3‐year follow‐up for all subjects reported recurrent strokes in 17.6% and 12.5% in patients with and without AF, respectively.^40^ Lower rate of recurrent stroke and higher rate of OAC initiation in patients receiving ILR compared with those receiving conventional cardiac rhythm monitoring have previously been reported in a single‐center study and 2 meta‐analyses.^7^, ^8^, ^41^ One should note that >20% of the present patients were taking OAC at baseline, mainly because most patients were enrolled before the publication of the results from NAVIGATE ESUS (New Approach Rivaroxaban Inhibition of Factor Xa in a Global Trial Versus ASA to Prevent Embolism in Embolic Stroke of Undetermined Source) and RE‐SPECT ESUS that did not show the superiority of direct OACs to aspirin for secondary prevention of ESUS.^39^, ^42^

The benefit of OAC in reducing stroke recurrence in patients with AF detected after stroke by ILR is unproven. In the recent ARTESIA (Apixaban for the Reduction of Thrombo‐Embolism in Patients with Device‐Detected Subclinical Atrial Fibrillation) and Non‐vitamin K Antagonist Oral Anticoagulants in Patients With Atrial High Rate Episodes trials including different cohorts with device‐detected subclinical AF (mainly patients who were stroke‐naïve), apixaban resulted in a lower risk of stroke or systemic embolism than aspirin, but edoxaban did not reduce the risk of composite events, including stroke, as compared with placebo or aspirin.^43^, ^44^ The combined analysis of these 2 trials showed a consistent reduction of ischemic stroke with direct OACs versus control (relative risk, 0.68 [95% CI, 0.50–0.92]) and an increase in major bleeding (relative risk, 1.62 [95% CI, 1.05–2.5]).^45^

One of the strengths of the present study was the participation of 12 countries, which might weaken regional imbalance. Another was the collection of real‐world data on the use of the ILR to monitor for AF after cryptogenic stroke. On the other hand, limitations include, first, the postmarket nonrandomized, observational design, which is subject to selection bias. The number of registered patients from 37 sites during >3‐year registration was unexpectedly small. The study design might also have caused underreporting of treatment strategies and outcomes. Second, due to the small sample size, a comparison between groups is likely not sufficiently powered to detect a difference; thus, the test result may turn out to be falsely negative. In addition, some patients seemed to have stroke of an unknown cause due to incomplete workup, not pure cryptogenic stroke, as described above. Such limitations might also affect the results. Third, the ILR used was a previous iteration yielding a higher rate of false‐positive alerts.^46^ Newer ILRs with remote reprogramming ability and dual‐stage algorithms that use artificial intelligence to decrease false‐positive alerts without missing clinically relevant events show higher true‐positive rates.^47^, ^48^ Fourth was the uneven follow‐up period between patients enrolled up to December 2017 (18 months) and those enrolled later (36 months), which possibly underestimated the AF detection rates and limited the collection of treatment strategies and outcomes in patients for a shorter period. Fifth, the analysis of AF predictors was performed only using components of CHA_2_DS_2_‐VASc score and country, although several other electrocardiographic, echocardiographic, and serologic biomarkers have been shown to be predictive of AF detection.

In conclusion, the rate of AF detection using ILR in patients with cryptogenic stroke from a real‐world global registry was similar to other studies in stroke populations monitored by ILRs, including CRYSTAL‐AF, STROKE‐AF and PER‐DIEM.^4^, ^5^, ^6^ Patients with detected AF more commonly initiated OAC than those without AF. Changes in treatment strategies such as OAC may have an impact on preventing stroke recurrence.

## Sources of Funding

Research funded by Medtronic Inc.

## Disclosures

Dr Toyoda reports honoraria from Bristol‐Myers‐Squibb, Bayer, Daiichi‐Sankyo, Janssen, and Otsuka. Dr Kusano reports honoraria from Bayer, Biotronik, Boston Scientific, Daiichi‐Sankyo, GE Precision Healthcare, and Medtronic. Dr Iguchi reports honoraria from Abbott, Chugai, Daiichi‐Sankyo, Novartis, Otsuka, Pfizer, Takeda, Tanabe‐Mitsubishi, and Sumitomo‐Pharma. Dr Ikeda reports honoraria from Bayer, and Daiichi‐Sankyo. Dr Morishima reports honoraria from Abbott and Daiichi‐Sankyo. Dr Tomita reports honoraria from Abbott, Bayer, Biotronik, Boehringer‐Ingelheim, Boston Scientific, Bristol‐Myers‐Squibb, Daiichi‐Sankyo, Fukuda Denshi, Japan Lifeline, and Medtronic. Dr Asano reports honoraria from Abbott and Medtronic. Dr Yamane reports honoraria from Daiichi‐Sankyo, Japan Lifeline, and Medtronic. Dr Kato reports honoraria from Abbott, Boston Scientific, Daiichi‐Sankyo, Japan Lifeline, and Medtronic. Dr Morita reports honoraria from Medtronic. Dr Hirano reports honoraria from Bayer, Boehringer‐Ingelheim, Daiichi‐Sankyo, Medtronic, Otsuka, and Pfizer. Dr Soejima reports honoraria from Abbott, Boehringer Ingelheim, and Medtronic. Dr Abe reports honoraria from Abbott, Boston Scientific, Fides‐One, Japan Lifeline, and Medtronic. Dr Yasaka reports honoraria from Bristol‐Myers‐Squibb, Boehringer‐Ingelheim, Bayer, Daiichi‐Sankyo, CSL Behring, and AstraZeneca. Dr Kasner reports honoraria from Bayer, Bristol‐Myers‐Squibb, Daiichi‐Sankyo, Medtronic, and WL Gore. Dr Natale reports honoraria from Abbott, Biosense Webster, Biotronik, Boston Scientific, and iRhythm. Dr Beinart reports honoraria from Abbott, Bristol‐Meyer Squibb, Janssen, and Zoll. Dr Amin reports honoraria from Alexion, Aseptiscope, AstraZeneca, Bayer, Blade Therapeutics, Bristol‐Myers‐Squibb, Dexcom, Eli Lilly, Ferring, Fulcrum Therapeutics, Gilead, GSK, HeartRite Humanigen, Jansen, National Institutes of Health/National Institute of Allergy and Infectious Diseases, NeuroRx, Nova Nordisk, Novartis, OctaPharma, Pfizer, Portola, PTC Therapeutics, Pulmotect, Reprieve, Renibus, Salix, Seres, Spero, and Takeda. E. Pouliot, Dr Franco, and Dr Hidaka are Medtronic employees and stakeholders. Dr Okumura reports honoraria from Boehringer‐Ingelheim, Bristol‐Myers‐Squibb, Daiichi‐Sankyo, J&J, and Medtronic. The remaining authors have no disclosures to report.
